# Efficient Differentiation of Embryonic Stem Cells into Hepatic Cells In Vitro Using a Feeder-Free Basement Membrane Substratum

**DOI:** 10.1371/journal.pone.0024228

**Published:** 2011-08-26

**Authors:** Nobuaki Shiraki, Taiji Yamazoe, Zeng Qin, Keiko Ohgomori, Katsumi Mochitate, Kazuhiko Kume, Shoen Kume

**Affiliations:** 1 Department of Stem Cell Biology, Institute of Molecular Embryology and Genetics, Kumamoto University, Honjo, Kumamoto, Japan; 2 Global Center of Excellence Program, Kumamoto University, Honjo, Kumamoto, Japan; 3 BM Matrix Laboratory, Environmental Health Sciences Division, National Institute for Environmental Studies, Ibaraki, Japan; Genome Institute of Singapore, Singapore

## Abstract

The endoderm-inducing effect of the mesoderm-derived supportive cell line M15 on embryonic stem (ES) cells is partly mediated through the extracellular matrix, of which laminin α5 is a crucial component. Mouse ES or induced pluripotent stem cells cultured on a synthesized basement membrane (sBM) substratum, using an HEK293 cell line (rLN10-293 cell) stably expressing laminin-511, could differentiate into definitive endoderm and subsequently into pancreatic lineages. In this study, we investigated the differentiation on sBM of mouse and human ES cells into hepatic lineages. The results indicated that the BM components played an important role in supporting the regional-specific differentiation of ES cells into hepatic endoderm. We show here that knockdown of integrin β1 (*Itgb1*) in ES cells reduced their differentiation into hepatic lineages and that this is mediated through Akt signaling activation. Moreover, under optimal conditions, human ES cells differentiated to express mature hepatocyte markers and secreted high levels of albumin. This novel procedure for inducing hepatic differentiation will be useful for elucidating the molecular mechanisms controlling lineage-specific fates during gut regionalization. It could also represent an attractive approach to providing a surrogate cell source, not only for regenerative medicine, but also for pharmaceutical and toxicologic studies.

## Introduction

The liver is an essential organ responsible for many kinds of metabolism, as well as for the storage of essential nutrients, the production and secretion of albumin (ALB), and biotransformation of xenobiotics. Biotransformation of xenobiotics usually results in their detoxification, but can also induce their bioactivation, if the metabolites produced are more toxic than their parent drug molecule. Although primary hepatocyte culture is a powerful tool for studying the bioactivation of xenobiotics, the hepatic function is inclined to be reduced in culture. In addition, bioactivation varies considerably depending upon the characteristics of the donors.

Embryonic stem (ES) cells are pluripotent cells derived from the inner cell mass of the blastocyst. *In vitro* approaches to inducing their differentiation into hepatic lineages involves the formation of embryoid bodies to mimic the inductive microenvironment required for liver organogenesis [1], [2], or their treatment with specific growth factors and cytokines critical for hepatocyte differentiation [3]. Co-cultivation of ES cells with embryonic mesenchymal cells has also been shown to direct ES cells towards a hepatic lineage [4], [5], [6]. Bone morphogenetic protein 4 (BMP4) was shown to induce the *in vitro* generation of mouse ES cell-derived hepatic cells [7]. The differentiation of human ES cells into hepatocytes has been shown using stage wise processes. Recent studies demonstrated that extrinsic signaling molecules Activin/Nodal direct differentiation into the definitive endoderm, fibroblast growth factor (FGF) plus BMP produce potentiated differentiation into hepatic lineage of human ES cells, retinoic acid (RA), Wnt 3a and dimethyl sulfoxide (DMSO) increase hepatic differentiation in human ES cells, then Hepatocyte growth factor (HGF) and Oncostatin M (OSM) promote maturation of hepatocytes [8], [9], [10], [11], [12], [13], [14]. Actually, these signaling molecules have been shown to function during embryonic development of the liver, therefore, ES cells are considered to recapitulate many aspects of normal developmental processes and serve as an attractive system for studies of developmental biology, applications for innovative drug screening strategies and regenerative medicine [15], [16], [17], [18].

We previously reported that the mesonephric cell line, M15, had the ability to induce ES cells into pancreatic or hepatic lineages *in vitro* [10], [11]. Under selective culture conditions, approximately 80% of human ES cells could be manipulated to differentiate intoα-fetoprotein (AFP)-positive cells, and 9% of total cells turned into ALB-positive cells; only the final stage of differentiation into regional-specific definitive endoderm required direct contact with M15 cells. The fact that even the fixed M15 cell layer retained the ability to induce pancreatic differentiation [10], suggested that the basement membrane (BM) components played a key role in guiding differentiation into regional-specific lineages of the definitive endoderm.

The BM has a highly integrated structure composed of extracellular matrix (ECM) molecules. The major components of most BMs are type IV collagen, laminins (LNs), entactin (nidogen) and heparin sulfate proteoglycans (HSPGs), such as perlecan. These molecules, together with secreted cytokines, are derived either from the epithelial cells, or from the surrounding mesenchymal cells, and are integrated into the structure of the BM [19], [20]. The molecules are then assembled to form an optimal extracellular environment for developing, regenerating or maturing cells. M15 cells expressed high levels of LN α5 (*Lama5*), relative to OP9 and PA6 cells, which showed no endodermal-inducing activities [21]. *Lama5* knockdown of M15 cells reduced their endodermal differentiation potential, thus indicating that *Lama5* is one of the components responsible for mediating the differentiation potency of M15.

The ECM is regarded as one of the important parameters for cell differentiation [22]. For human ES cells, which predominantly express integrin α6β1, recombinant LN332, LN511 and LN111 were shown to serve as good substrates to expand undifferentiated human ES cells [23]. Three dimensional scaffolds such as matrigel or collagen were shown to be effective in inducing hepatocyte differentiation from human ES cells when added with hepatocyte growth factor *in vitro* [24], [25]. Other three dimensional fibrous scaffolds and spheroid foams made from polyesters have been shown to support hepatic differentiation of mouse ES cells in the presence of specific growth factors [26], [27].

We recently reported a novel approach for directing ES cell differentiation on a solid environment of BM, without the need for an M15 feeder layer [21]. We described a synthesized BM (sBM) substratum using an HEK293 cell line stably expressing human recombinant LN-511 (rLN511 sBM), into which rLN-10 cells [28] secreted and integrated the BM components. Using this sBM substratum, mouse ES cells or induced pluripotent stem (iPS) cells were differentiated into definitive endoderm and further into pancreatic lineages [21]. In this study, we investigated the ability of the sBM substratum to also support the hepatic differentiation of both mouse and human ES cells.

## Results

### ES cells grown on rLN511-sBM substratum could differentiate into AFP-expressing hepatic progenitor cells and functional ALB-secreting hepatocytes

We thus prepared a sBM substratum of human recombinant LN511 (rLN511 sBM), into which rLN-10 cells [28] secreted and integrated the BM components.

**Figure 1:**
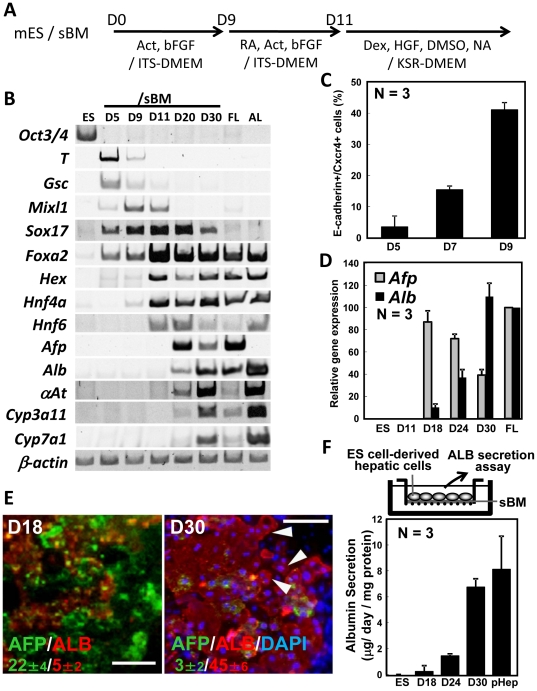
Differentiation of mouse ES cells into definitive endoderm and cells of the hepatic lineage on sBM. (A) Schematic of the experimental procedure. Mouse ES cells (mES) were differentiated on day (D) 0–9 in serum-free DMEM supplemented with ITS, 20 ng/ml activin and 50 ng/ml bFGF. RA 1 µM was added to the differentiation medium at D9–11. At D11, the supplements were changed to 1 µM Dex, 10 ng/ml HGF, 1% DMSO and 1 mM NA in KSR-DMEM. (B) Lineage-specific molecular marker expression in undifferentiated ES cells (ES), differentiated ES cells grown on sBM on D5, 9, 11, 20 and 30, Fetal liver (FL) and Adult liver (AL). (C) Flow cytometry of differentiated ES cells on sBM on D5, D7 and D9. Forty percent of total ES cell-culture area consisted of E-cadherin^+^/CXCR4^+^ definitive endoderm cells. Values represent means ± S.E.M (N = 3). (D) Relative expression levels of hepatic marker genes, *Afp* (*alpha fetoprotein*, gray bars) and *Alb* (*albumin*, black bars) in differentiated ES cells at D11, D18, D24, D30 and in fetal liver were quantified by real-time PCR analyses. The expression levels were normalized to that of *β-actin*. FL: E12.5 fetal liver. Values represent means ± S.E.M (N = 3). (E) ES cells differentiated for 18 or 30 days were stained for AFP (Alpha-fetoprotein; green) or ALB (Albumin; red), and counterstained with DAPI (blue). Percentages indicated in green or red are the positive cells for AFP or ALB, respectively. Arrowheads indicate binuclear hepatocyte-like cells. Scale bars  = 100 µM. Values represent means ± S.E.M (N = 3). (F) A diagram of the ES cell-derived hepatic cells grown on sBM and samples used for Albumin (ALB) secretion assay. Albumin secretion by mouse ES cell-derived hepatic cells was assayed by ELISA. The differentiation medium was changed to fresh medium 24 hrs before the assay. The amount of ALB released from the ES cell-derived hepatic cells into the medium per 24 hrs per mg protein was measured on day (D) 11, D18, D24 and D30 of differentiation on sBM. Albumin secretion level was normalized with total protein. Values represent means ± S.E.M (N = 3).

For hepatic differentiation, the mouse SK7 ES cell line was seeded onto the rLN511 sBM substratum and cultured with serial change of media shown in Figure 1A. The expressions of mesenendoderm markers (*T* and *Gsc*), definitive endoderm markers, (*Mixl1*, *Sox17* and *Foxa2*) and hepatic endoderm markers (*Hex*, *Hnf4a*, *Hnf6*) were observed by day 11 (D11) of differentiation. The hepatic progenitor markers (*α-fetoprotein*; *Afp*) were expressed from D20. Markers that were specific for mature hepatocytes, such as *albumin* (*Alb*), *α1-antitrypsin* (*αAT*), *Cyp3a11* and *Cyp7a1*, were expressed from D20 or D30 (Fig. 1B). The differentiation of ES cells into the definitive endoderm (DE) was quantified by flow cytometry, showing an increase with time from day 5 (D5) to D9 of differentiation, and that 40% of cells differentiated into E-cadherin-positive, CXCR4-positive DE cells on D9 (Fig. 1C). Negative control stained with isotype controls of the CXCR4 and the E-Cadherin was confirmed to give no signals (Fig. S1). Quantitative reverse transcription-polymerase chain reaction (RT-PCR) analysis revealed that *Afp* was expressed at a high level on D18, and decreased thereafter, while expression of the hepatocyte marker, *Alb*, was detectable from D18, and increased thereafter. The levels of *Afp* and *Alb* transcripts on differentiation D18 and D30, respectively, were comparable to the levels in fetal liver (FL) (Fig. 1D). Immunocytochemical analyses indicated that both AFP and ALB proteins were present in the cytoplasm (Fig. 1E). Some of the ALB-positive cells were binuclear (Fig. 1E, arrowheads), which is a characteristic of mature hepatocytes. Quantitative measurements revealed that approximately 22±4% of cells were AFP-positive at D18, which decreased to 3±2% at D30, whereas 5±2% of cells were ALB-positive at D18, which increased to 45±6% at D30 (Fig. 1E). ALB secretion could be detected from D18, which increased with days and reached a rate of 6.8 µg/day/mg protein at D30. Taking into account that 45±6% cells expressed ALB, the ALB secretion ability of the ALB-positive cells was approximately comparable to that of the adult mouse primary hepatocytes (Fig. 1F, pHep).

Here, we cultured ES cells on sBM and differentiation was done under novel culture conditions compared to our previous report [11]. To clarify the differences, we compared ALB secretion levels yielded among different differentiation conditions. Differentiation performed on sBM yielded a 1.5-fold increase in ALB secretion level compared to that grown on M15 cells. Addition of RA, nicotinamide (NA) and DMSO further yielded a 21-fold increase in ALB secretion and yielded Albumin secretion 6.8 µg/day/mg protein (Table S2).

Taken together, the above results indicated that the sBM substratum served as a suitable solid environment of extracellular matrix structure with the potential to guide mouse ES cell differentiation into the hepatic lineage, secreting ALB at a level comparable to that of the mature adult hepatocytes.

### Differentiation of ES cells on sBM is mediated through integrin β1

**Figure 2:**
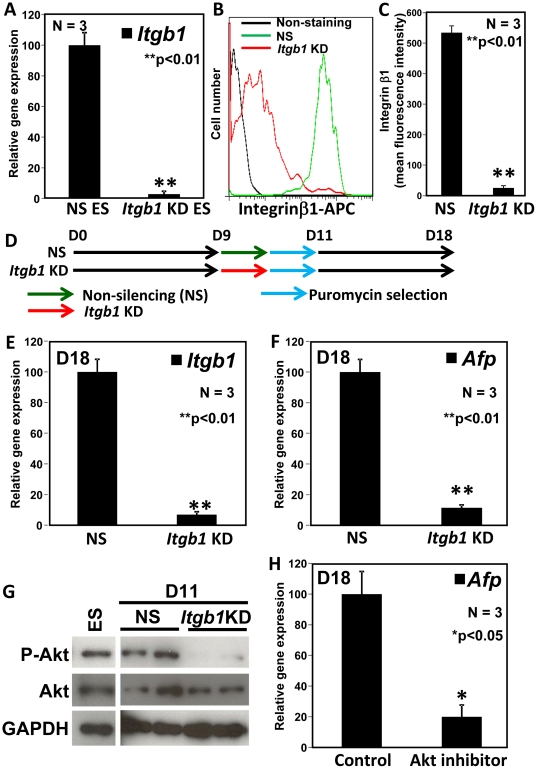
Differentiation of ES cells into hepatic lineages on sBM is mediated through integrin β1. (A) Real-time PCR analysis of *Itgb1* expression in NS or *Itgb1* KD ES cells. Significant differences were observed (**p<0.01) versus NS. Values represent means ± S.E.M (N = 3). (B) Flow cytometry analysis of Itgb1 expression in NS (green line) or *Itgb1* KD (red line) ES cells. Black line indicated unstained samples. (C) Mean fluorescence intensity of Itgb1 in NS or *Itgb1* KD undifferentiated ES cells. Significant differences were observed (**p<0.01) versus NS. Values represent means ± S.E.M (N = 3). (D) ES cells were cultured as described in Fig. 1 and non-silencing (NS) or *Itgb1*-knockdown (*Itgb1* KD) lentivirus-containing medium was added on day (D) 9. Puromycin selection from D10 to D11 was used to remove uninfected cells. Cells were analyzed on D18. (E, F) Real-time PCR analysis of *Itgb1* and *Afp* expression in NS or *Itgb1* KD ES cells. Total RNA was extracted on D18. *Itgb1* and *Afp* expression were normalized to that of *β-actin*. Significant differences were observed (**p<0.01) versus NS. Values represent means ± S.E.M (N = 3). (G) Western blot analysis of phospho-Akt, Akt and GAPDH in the *Intgb1* KD or control (NS) cells. GAPDH is used as an internal control for total proteins. The phosphorylation level of Akt was significantly reduced in *Intgb1* KD but not in NS cells (N = 2). (H) Real-time PCR analysis of *Afp* expression in Akt inhibitor treated cells on D18. Significant differences were observed (*p<0.05) versus NS. Values represent means ± S.E.M (N = 3).

We previously showed that BM components play an important role in the differentiation of definitive endoderm lineages, and that *Lama5* is one of the key guiding signals in the sBM [21]. Also, we showed that *Lama5* signaling in ES cells is mediated through integrin-β1 (Itgb1), which is known to interact with LNs. We performed *Itgb1* knockdown in differentiating ES cells to determine if *Itgb1* also mediates extracellular guiding signals in ES cells in the case of hepatic differentiation. To evaluate the knockdown efficiency, we transfected *Itgb1*-knockdown lentivirus (*Itgb1* KD) to undifferentiated ES cells and established an *Itgb1*KD ES cell line. *Itgb1*KD ES cells showed a decreased *Itgb1* expression level, compared to non-specific shRNA transfected (NS) ES cells (Fig.2A). The decrease in Itgb1 protein level was confirmed by flow cytometry analysis, and the mean fluorescence intensity decreased to less than 1/20 fold (Fig. 2B, C). Since *Itgb1*KD ES cells showed a decreased attachment to the plate, we then added the *Itgb1*KD to ES cell cultures on D9, followed by puromycin selection for one day on D10 to enrich lentivirus-transduced cells (Fig. 2D). The dose response for assessing cell death by graded concentrations of puromycin was performed empirically to ensure the enrichment of the *Itgb1*KD transfected ES cells (Fig. S2). The expression level of *Itgb1* transcripts was analyzed on D18, and hepatic lineage differentiation was assayed by detection of *Afp* transcripts. *Itgb1* KD cells expressed a decreased level of *Itgb1* transcripts approximately 1/10-fold of the non-silencing negative control samples (NS) (Fig. 2E), and yielded a reduced level of *Afp* transcripts approximately 1/10-fold of the control (Fig. 2F).

The serine-threonine kinase Akt (protein kinase B / PKB) was known as one of a downstream molecule of integrin signals [29]. Western blot analysis revealed that phosphorylation of Akt was inhibited in *Itgb1* KD cells (D11) (Fig. 2G), thereby suggesting that Akt lying downstream of Itgb1 signaling here. A cell-permeable potent Akt inhibitor benzimidazole compound [30] was added from D9 to D18, and *Afp* transcript level decreased to 1/5-fold of the control (Fig. 2H). These results suggest that the extracellular signals from the sBM guiding ES cells differentiation into the hepatic lineage is transduced through the Itgb1-Akt signaling pathway.

### Human ES cells could differentiate into hepatic lineages on sBM

**Figure 3:**
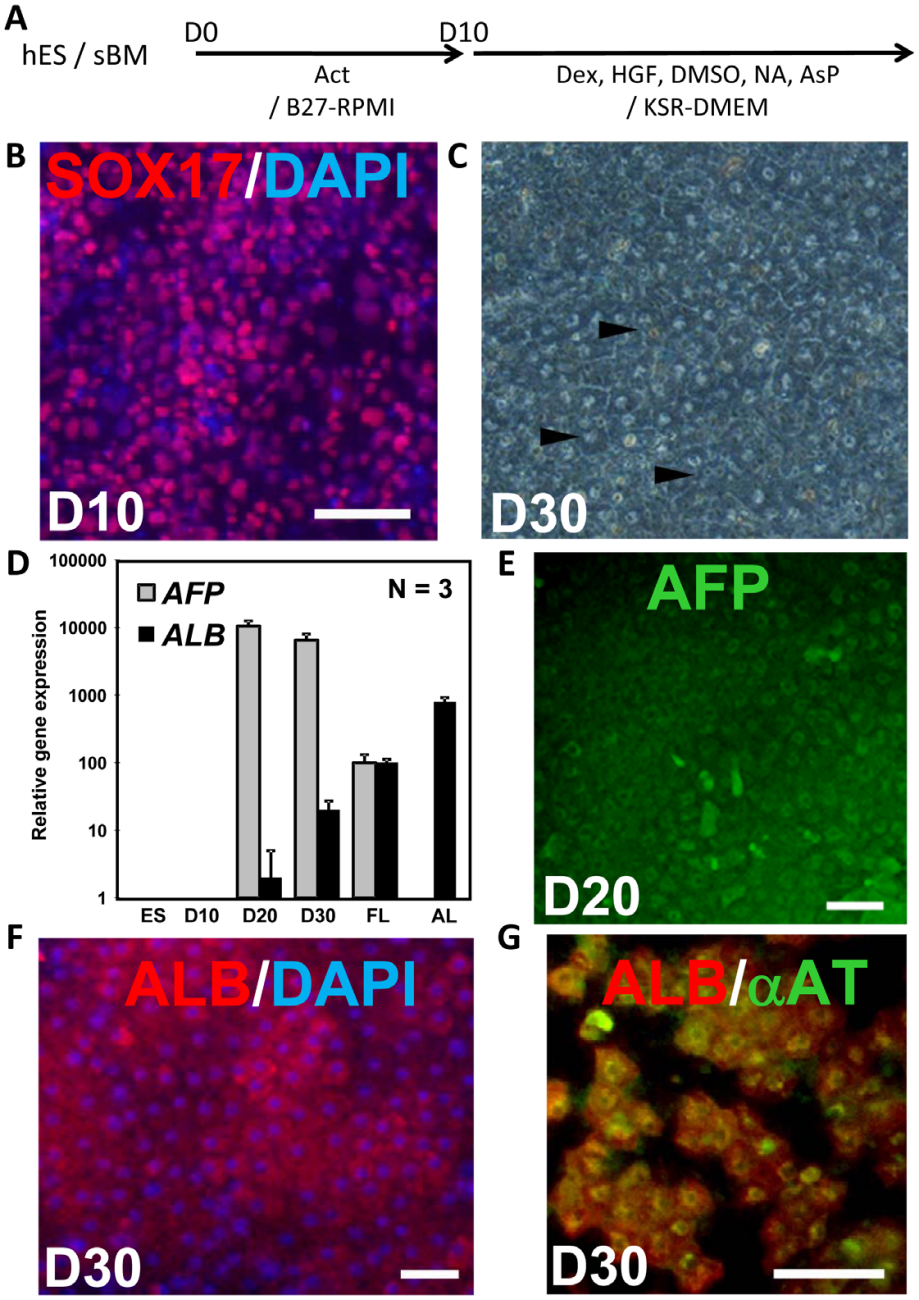
Human ES KhES-3 cells differentiated into hepatic cells on the sBM substratum. (A) Human ES cells were differentiated at D0–10 in serum-free medium supplemented with 100 ng/ml activin. At D10, the supplements were switched to 1 µM Dex, 10 ng/ml HGF, 0.5% DMSO, 0.5 mM NA and 0.2 µM AsP. (B) KhES-3 cultured for 10 days were stained for Sox17 (red), and counterstained with DAPI (blue). Scale bars  = 100 µM. (C) KhES-3 cells on D30 (unstained). Polygonal cells were observed (arrowheads). (D) Relative expression levels of *Afp* (*alpha fetoprotein*; gray bars) and *Alb* (*albumin*; black bars) in differentiated KhES-3 cells at day (D) 10, D20, and D30, and in human fetal (FL) and adult liver (AL) were quantified by real-time PCR analyses. The expression levels were normalized to that of glyceraldehyde phosphate 3-dehydrogenase (*GAPDH*). Values represent means ± S.E.M (N = 3). (E, F) KhES-3 cells cultured for 20 or 30 days were stained for AFP (green) (E) or ALB (red) (F). Nuclei were stained with DAPI (blue). Scale bars  = 100 µM. (G) KhES-3 cells cultured for 30 days were stained for both α-antitrypsin (green) and ALB (red). Scale bars  = 100 µM.

We also determined the ability of the sBM to allow the differentiation of human ES cells. The human KhES-3 ES cell line was cultured on sBM, and the expression of the definitive endoderm marker, Sox17, was observed on D10 differentiation (Fig. 3A, B). By D30 of differentiation, KhES-3 cells had formed a monolayer of polygonal cells, including some binuclear cells, which is a characteristic of mature hepatocytes (Fig. 3C, arrowheads). The expression level of *Afp* transcripts was high on D20, but had decreased on D30. *Alb* transcripts were detected from D20, and had increased on D30, but the level remained lower than that in human fetal (FL) or adult liver (AL) (Fig. 3D). Immunocytochemical analyses show AFP expression on D20 and ALB cytoplasmic staining on D30 (Fig. 3E, F). The hepatic cells expressing ALB were also positive for α-antitrypsin. These results demonstrate that sBM allowed the differentiation of human ES cells into hepatic lineages.

### Human ES cells grown on sBM differentiated in vitro into hepatic cells expressing mature hepatocyte markers and secreting albumin

**Figure 4:**
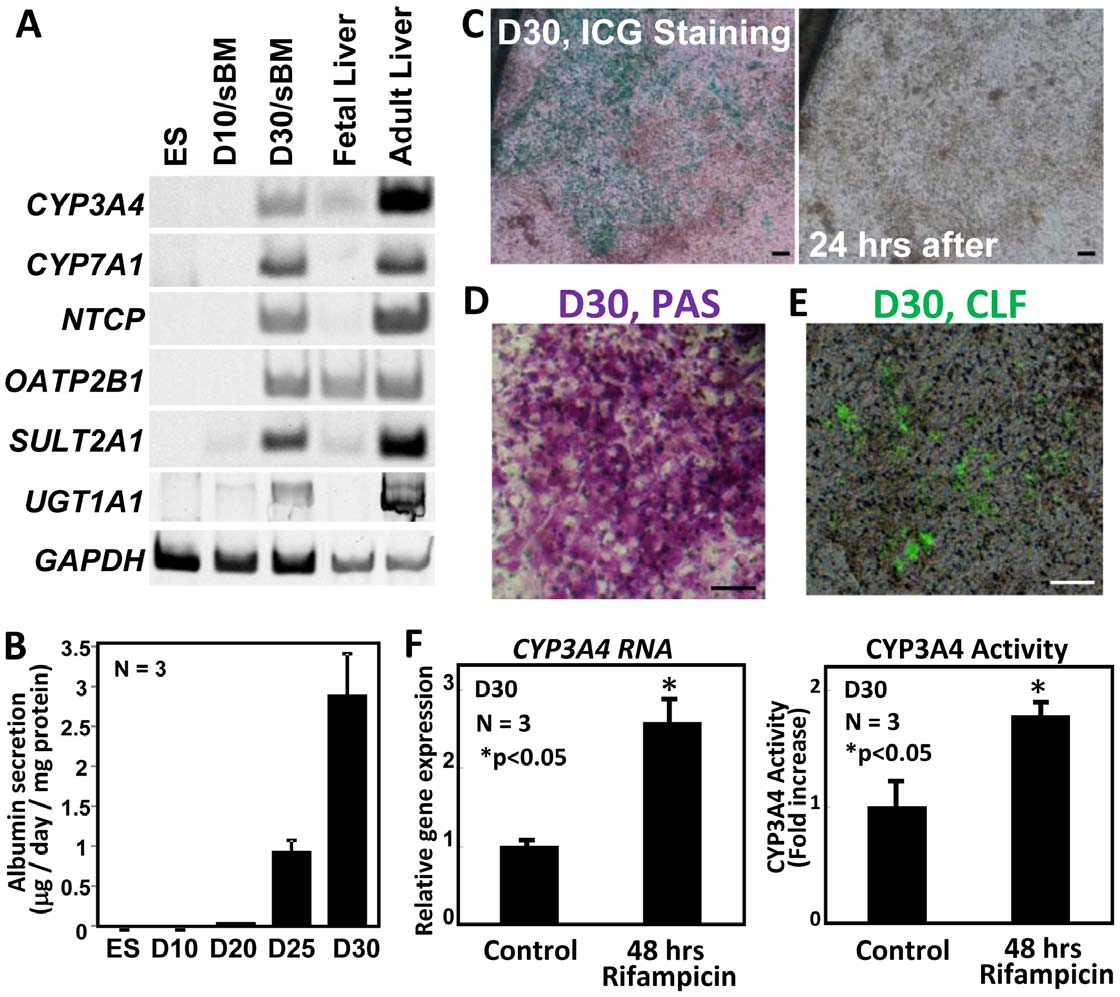
Expression of functional hepatocyte markers in differentiated khES-3 cells. (A) Expression of *CYP3A4, CYP7A1, NTCP, OATP2B1, SULT2A1, UGT1A1* and *GAPDH* in differentiated KhES-3 cells at D10, and D30, in human fetal and adult liver. (B) Albumin secretion by human ES cell-derived hepatic cells was assayed by ELISA. The differentiation medium was changed to fresh medium 24 hrs before the assay. The amount of albumin released from the ES cell-derived hepatic cells into the medium per 24 hrs per mg protein was measured at D10, D20, D25 and D30 of differentiation on sBM. Values represent means ± S.E.M (N = 3). (C) ICG-positive cells were detected at differentiation D30. (D) PAS assay of D30 differentiated ES cells indicated numerous hepatocytes within colonies exhibiting cytoplasmic glycogen storage. (E) CLF-positive cells were detected at differentiation D30. Scale bars  = 100 µM (C–E). (F) Treatment with rifampicin for 48 hrs induced both *CYP3A4* transcripts (left panel) and CYP3A4 enzyme activity (right panel) in KhES-3-derived hepatic cells. Significant differences were observed (*p<0.05) versus control. Values represent means ± S.E.M (N = 3).

Regarding the expression of other mature hepatocyte markers, substantial levels of Cytochrome P450 3A4 (CYP3A4), Cytochrome P450 7A1 (CYP7A1), Na+-taurocholate cotransporting polypeptide (NTCP), Organic anion transporting polypeptides 2B1 (OATP2B1), Sulfotransferase 2A1 (SULT2A1) and UDP glucuronosyltransferase 1A1 (UGT1A1) transcripts were detected in differentiated KhES-3 cells at D30 (Fig. 4A). Except OATP2B1, these markers were absent from fetal liver, thereby indicating that the ES cell-derived hepatic cells expressed mature hepatocyte markers. ALB secretion was detected in human ES cell-derived hepatocytes from D20, and reached a peak of approximately 2.9 µg/day/mg protein at D30 (Fig. 4B). Hepatocytes derived from human ES cells incorporated indocyanine green (ICG) (Fig. 4C, left panel), which was excluded after 24 hrs (Fig. 4C, right panel). These cells were also periodic acid-Schiff (PAS)-positive, indicating cytoplasmic glycogen storage (Fig. 4D). The primary bile acid analog cholyl lysyl fluorescein (CLF), which is a substrate for the excretion transporter [31], was rapidly taken up and transported into bile canaliculi, indicating that functional transporters were expressed in the human ES cell-derived hepatocytes (Fig. 4E). These cells expressed high levels of CYP3A4 transcripts (Fig. 4F, left panel) and showed CYP3A4 enzymatic activity (Fig. 4D, right panel), both of which were induced by rifampicin treatment.

Overall, these results indicate that human ES cells grown on sBM were able to differentiate into hepatic cells expressing mature hepatocyte markers and secreting albumin.

## Discussion

Previous studies suggested that the addition of soluble growth factors was sufficient to support the differentiation of ES cells into definitive endoderm. However, direct contact between ES cells and M15 cells was necessary for the progression of regional-specific differentiation into endodermal tissues [10]. We also reported the development of a sBM by culturing HEK293 cells overexpressing rLN511 (LNα5, β1 and γ1) (rLN511 sBM), which could be used to allow the subsequent differentiation of ES cells or iPS cells into pancreatic lineages, and further into insulin-expressing endocrine cells [21]. The pancreatic differentiation signal was mediated by the components of the BM, including LN α5 and HSPGs. In the current study, we demonstrated that the sBM was also able to support the hepatic-lineage differentiation of ES cells, and that *Itgb1* plays a role in mediating hepatic differentiation. The efficacy of hepatic differentiation on sBM was high, and the derived differentiated cells were able to further differentiate into hepatic cells expressing mature hepatocyte markers and secreting ALB. This procedure was applicable to both mouse and human ES cells. The ES cell-derived hepatic cells were induced to sequentially express *T*, *Gsc*, *Mixl1*, *Sox17*, *Foxa2*, *Hex*, *Hnf4a*, *Hnf6*, *Afp* and *Alb*, recapitulating the normal developmental processes. These ES cell-derived hepatic cells expressed mature hepatocyte markers *αTT* , *Cyp3a11* and *Cyp7a1*. These results indicate that although the ES cell-derived hepatic cells expressed *Alb* transcripts at a much lower level, they showed transcriptional profiles of the mature hepatocytes. Human ES-derived hepatic cells exhibited several mature hepatic profiles, such as ALB secretion, uptake and export of ICG and CLF, glycogen storage, and CYP3A4 metabolic enzyme activity.

The *Itgb1* KD experiment demonstrated that the guiding signal from sBM was transduced through Itgb1, which functions as a key component in the differentiation of ES cells into the hepatic lineage. Integrins bind the ECM as heterodimers consisting of different combinations of α and β subunits. Itgb1 is a cell surface receptor that mediates cell-matrix and cell-cell interactions. Epithelial cells derived from endodermal gut tubes, such as lung, esophagus, trachea, stomach, hindgut and urinary bladder, all express Itgb1 [32]. During hepatic development, Itgb1 is expressed in the mouse early foregut endoderm at embryonic development day (E)9.5 exclusively in *Afp*-expressing cells, and later at E17.5 in blood vessels in sinusoidal structures [33]. *Itgb1* mutant mice were reported to die early in development, prior to the onset of hepatogenesis [34], while analysis of chimeric mice, generated by combining wild-type embryos with *Itgb1* mutant ES cells, revealed that cells lacking Itgb1 were incapable of colonizing the liver. This result implies that there is a cell autonomous requirement for Itgb1 in defining or maintaining the hepatic cell lineage [34]. Moreover, Itgb1 levels in Smad2^+/-^ and Smad3^+/-^ mutant mice, in which liver architecture was disrupted and the developing hepatic cells appeared unable to generate normal cell-cell adhesions, were 10% of control levels [35]. Interestingly, this phenotype could be rescued by HGF in culture, and the authors suggested that the HGF and Smad signaling pathways might converge on Igtb1 to promote hepatic development [35].

Itgb1 is implicated in mediating cell behavior in hepatocytes, including the attachment of hepatocytes to hepatic ECM, thereby maintaining hepatocyte survival [36]. Transforming growth factor β has been reported to control the directional migration of hepatocytes by modulating the expression of Itga5b1 expression and of its ligand, fibronectin [37], while functional blockade of Itga5b1 induced cell scattering and spreading [38]. Integrin expression thus seems to be important for the regulation of hepatocyte motility in response to cytokine signaling during embryonic liver development.

The major ECM components LN, collagen IV, HSPG, nidogen and fibronectin are expressed in the intrahepatic biliary duct from E13.5 throughout development and after birth [33], and laminin and collagen types I and IV facilitated the hepatic differentiation of a subpopulation of hepatic stem cells isolated by flow cytometry [39]. Overall, the BM components appear to be important for hepatic development. LN expression in humans is observed during embryonic and fetal development, similar to the situation in mice [40].

This study demonstrates the importance of the BM components in the differentiation of mouse and human ES cells into the hepatic lineage. From undifferentiated human ES cells grown on one 90 mm-dish (1×10^7^ cells), approximately 2×10^8^ cells of human ES cell-derived hepatic cells can be generated using the present sBM system after 30 days culture. Therefore, it is feasible to utilize this culture procedure to provide a surrogate cell source for regenerative medicine, as well as for pharmaceutical and developmental biology studies.

## Materials and Methods

### ES Cell lines

The mouse ES cell line, SK7 [10] was maintained on mouse embryonic fibroblast (MEF) feeder cells in Glasgow minimum essential medium (Sigma-Aldrich, St. Louis, MO) supplemented with 1,000 U/ml leukemia inhibitory factor (Chemicon, Tmmecula, CA), 15% KSR (Invitrogen, Carlsband, CA), 1% fetal bovine serum (FBS; Hyclone, Logan, UT), 100 mM nonessential amino acids (NEAA; Invitrogen), 2 mM L-glutamine (L-Gln; Nacalai Tesque, Kyoto, Japan), 1 mM sodium pyruvate (Invitrogen), 50 U/ml penicillin and 50 mg/ml streptomycin (PS; Nacalai Tesque) and 100 µM β-mercaptoethanol (βME; Sigma-Aldrich) as described previously [10], [41].

Human ES cells (KhES-3) [42] were from Dr. Norio Nakatsuji and Dr. Hirofumi Suemori (Kyoto University, Kyoto, Japan) and were used in accordance with the human ES cell guidelines of the Japanese government. This human ES work was approved by Kumamoto University institutional review board. Undifferentiated human ES cells were maintained as described previously [11].

### Growth factors and inhibitors

Reagents were purchased and used at the designated concentrations as follows: recombinant human activin-A (R&D Systems, Minneapolis, MN), 20 ng/ml (for mouse ES cells) or 100 ng/ml (for human ES cells); recombinant human bFGF (Peprotech, Rocky, NJ), 50 ng/ml; Retinoic acid (RA, Sigma-Aldrich), 10^−6^ M; recombinant human HGF (Peprotech), 10 ng/ml; Dexamethasone (Sigma-Aldrich), 1 µM; Dimethylsulfoxide (DMSO, Sigma-Aldrich), 1% (for mouse ES cells) or 0.5% (for human ES cells); Nicotinamide (NA, Sigma-Aldrich), 1 mM (for mouse ES cells) or 0.5 mM (for human ES cells); ascorbic acid (AsP, Sigma-Aldrich), 0.2 µM; Y-27632 (Rho-associated kinase inhibitor, Wako Chemical, Osaka, Japan), 10 µM. Akt inhibitor IV (Calbiochem, Darmstadt, Germany) 1 µM.

### sBM preparation

The sBM was prepared as described previously [21]. Mochitate, K. Method of preparing BM, method of constructing BM specimen, reconstituted artificial tissue using the BM specimen and process for producing the same. US Patent number 7,399,634 and 7,906,332. Human LN-511 (rLN-10) derived from 293 cells was a kind gift from Dr. Masayuki Doi and Dr. Karl Tryggvason at Karolinska Institute, Sweden [28].

### Differentiation of ES cells on sBM substratum into hepatic lineage

The sBM substratum was stored at −75°C and gently thawed in a refrigerator one night before use. ES cells grown on MEF were dissociated and plated at a 5,000 cells per sBM substratum (12-well culture insert, surface area  = 0.9 cm^2^). For hepatic differentiation, ES cells were cultured in 4,500 mg/l glucose DMEM containing insulin (10 mg/l), transferrin (5.5 mg/l), sodium selenite (6.7 mg/ml) ITS (Invitrogen), ALBUMAX II (2.5 mg/ml) (Invitrogen), NEAA, L-Gln, PS and βME (ITS-DMEM), supplemented with activin A (20 ng/ml) and bFGF (50 ng/ml) at D0–D9, and RA (10^−6^ M) was added from D9–D11. This was then switched to 2,000 mg/l glucose DMEM containing 10% KSR, NEAA, L-Gln, PS and βME (KSR-DMEM), supplemented with HGF (10 ng/ml), Dex (1 µM), DMSO (1%), and NA (1 mM) at D11, then cultured up to D30.

Human KhES-3 cells were pre-treated with Y-27632, a potent Rho-kinase inhibitor, for 12 hrs, then dissociated using 0.25% trypsin-EDTA, and plated at 50,000 cells per sBM substratum. ES cells were cultured in RPMI-1640 (Invitrogen) supplemented with activin A (100 ng/ml), B27 supplement (2%) (Invitrogen), NEAA, L-Gln, PS and βME for 10 days. They were then switched to hepatic differentiation medium: KSR-DMEM supplemented with HGF (10 ng/ml), Dex (1 µM), DMSO (0.5%), NA (0.5 mM), and AsP (0.2 µM) at D10 then cultured up to D30. Medium was replaced every 2 days with fresh differentiation medium supplemented with growth factors.

### Hepatocyte isolation and culture

Hepatocytes were isolated from the whole liver of an adult ICR mouse (male, 7–8 weeks old) by the two-step liver perfusion method of Seglen [43]. Cell viability was determined by trypan blue exclusion, and cells used were obtained with more than 50% viability. The culture medium used were consisted of DMEM supplemented with ITS, PS, L-Gln, 7.5 mg/L hydrocortisone (Sigma), 50 µg/L epidermal growth factor (Peprotech), 60 mg/L proline (Sigma), 50 µg/L linoleic acid (Sigma), 0.1 µM CuSO_4_5 H_2_O (Sigma), 50 pM ZnSO_4_7 H_2_O (Sigma) [44].

### Reverse-transcription polymerase chain reaction (RT-PCR) and real-time PCR analysis

RNA extraction, RT, PCR analysis, and real-time PCR analysis were performed as described previously [10], [11]. Human fetal (22–40 weeks old) and adult (51 years old) liver total RNAs were purchased from Clontech Laboratories, Inc. The primer sequences for each primer set are shown in Table S1. The PCR conditions for each cycle were: denaturation at 96°C for 30 sec, annealing at 60°C for 2 sec, and extension at 72°C for 45 sec. RT-PCR products were separated by 5% non-denaturing polyacrylamide gel electrophoresis, stained with SYBR Green I (Molecular Probes, Eugene, NY), and visualized using a Gel Logic 200 Imaging System (Kodak, Rochester, NY). The real-time PCR conditions were as follows: denaturation at 95°C for 15 sec, annealing and extension at 60°C for 60 sec, for up to 40 cycles. Target mRNA levels were expressed as arbitrary units, and were determined using the standard curve method.

### Immunocytochemistry

For whole-mount immunocytochemical analysis, ES cell cultures were fixed in 4% paraformaldehyde in phosphate-buffered saline (PBS) for 30 min, followed by permealization with 0.1% Triton-X (Nakalai Tesque) in PBS for 10 min at room temperature, rinsed several times with PBS then incubated with diluted antibody in 20% Blocking One (Nacalai Tesque) in PBST (0.1% Tween-20 in PBS) in a humidified chamber overnight at 4°C. Cells were washed in PBST, and incubated with secondary antibody in 20% Blocking One for 2 hr at room temperature in the dark. After washing off the secondary antibody in PBST, cells were counterstained with 6-diamidino-2-phenylindole (DAPI) (Roche Diagnostics, Indianapolis, IN). The following antibodies were used as first antibodies: rabbit anti-AFP (Dako, Glostrup, Denmark), goat anti-ALB (Sigma-Aldrich), rabbit anti-α-1-antitrypsin (Sigma-Aldrich) and goat anti-Sox17 (R&D systems). Secondary antibodies used were Alexa 568-conjugated and Alexa 488-conjugated antibodies (Invitrogen).

### Gene silencing

Expression arrest control shRNA (Open Biosystems, #RHS4080) or *Itgb1* shRNA (Open Biosystems, #RMM3981-97055034) lentiviral vectors carrying puromycin-resistance genes were used for *Itgb1*-knockdown assays. For virus preparation, HEK293-FT cells (Invitrogen) were plated the day before transfection. After overnight culture, the cells were transfected with lentiviral vectors and ViraPower Lentiviral Packaging Mix (Invitrogen) using FuGENE6 Transfection Reagent (Roche Diagnostics) for 24 hours, and viral supernatants were collected. Titers of virus were determined using a Lenti-X-qRT-PCR titration kit (Clonetech). The M.O.I used was 500.

### Flow cytometry analysis

The following antibodies were used: biotin-conjugated anti-Integrin β1 monoclonal antibody (mAb) (BD Biosciences Pharmingen), Streptavidin-conjugated APC (BD Biosciences Pharmingen). The stained cells were analyzed with a FACS Canto (BD). Data were recorded with the BD FACS Diva Software program (BD) and analyzed using the Flowjo program (Tree Star).

### Western Blot

NS or *Intgb1* KD cells were homogenized in SDS sample buffer (62.5 mM Tris-HCl, 10% glycerol, 2% SDS, pH 6.8). After centrifugation, the supernatants were collected and used as total protein extracts. Total proteins were subjected to 10% SDS-PAGE and transferred to polyvinylidene difluoride membranes. The membranes were incubated with rabbit anti-GAPDH antibody (Cell Signaling Technology (CST), Beverly, MA) at 1∶1000 dilutions, rabbit anti-Akt antibody (CST) at 1∶1000 dilution, or rabbit anti-Phospho Akt antibody (CST) at 1∶1000 dilution. Horseradish-peroxidase-conjugated anti- rabbit Ig (CST) was used as a secondary antibody at 1∶20,000 dilutions, and chemiluminescent signals were detected with ECL Plus western blotting detection reagents (GE Healthcare, Chalfont Saint Giles, UK).

### Albumin secretion assay

Culture medium was replaced 1 day prior to the assay with 0.5 ml/1.0 ml fresh medium in the upper/lower compartment of sBM (12-well culture insert, surface area  = 0.9 cm^2^). Conditioned medium was harvested 24 hrs later and assayed for albumin secretion using an enzyme-linked immunosorbent assay (ELISA) kit (Bethyl, Montgomery, TX). Albumin secretion levels were normalized with total protein of differentiated ES cells at each sampling point. The protein amounts were calculated using Bio Rad Protein Assay Kit (Bio Rad, Hercules CA).

### Indocyanine green (ICG) test

ICG (Daiichi-Sankyo Pharma, Tokyo, Japan) was diluted in the above culture medium to a final concentration of 1 mg/ml. The ICG test solution was added to the differentiated ES cells on D30, and incubated at 37°C for 30 mins. Cells were washed three times with PBS, and the cellular uptake of ICG was then examined by microscopy.

### PAS analysis

The cultured cells were fixed in 3.3% formalin for 10 mins, and intracellular glycogen was stained using a PAS staining solution (Muto Pure Chemicals, Tokyo, JAPAN), according to the manufacturer's instructions.

### Cholyl lysyl fluorescein (CLF) staining

The cells were rinsed with PBS, and 5 µM CLF (BD Biosciences, San Diego, CA) in medium was added. After incubation for 30 mins, the cell cultures were rinsed with PBS. Cell morphology and CLF accumulation in bile canaliculi-like structure were assessed by phase-contrast and fluorescence microscopy.

### CYP3A4 activity

To check the inducibilities of cytochrome P450 activities in response to inducers, we used a P450-Glo™ CYP Assay Kit (Promega, Madison, WI). The differentiated ES cells were treated with 10 µM rifampicin as an inducer of CYP3A4. Medium containing the inducer was changed every 24 hrs. The medium was changed 48 hrs after treatment, and the appropriate luminogenic CYP substrates were added [Luciferin-Penta Fluoro Benzyl Ether (PFBE) for CYP3A4]. The cells were incubated at 37°C for 3 hrs, and the supernatants were then mixed with equal amount of Detection Reagent, according to the manufacturer's instructions (Promega). The luminescence was measured using a GloMax 96 microplate luminometer (Promega), using luminometer settings according to the manufacturer's instructions.

## Supporting Information

Figure S1 **Flow cytometry of differentiated ES ells grown on sBM on D9.** Left panel: unstained negative control. Right panel: 40% of total ES cells were E-cadherin+/CXCR4+ definitive endoderm cells.
(TIF)

Figure S2 **Dose dependent death of differentiated ES cells by puromycin.** ES cells were cultured as described in Fig. 1 and 0.5, 1.0 or 1.5 µg/ml puromycin were added from D10 to D11. On D11, cells were harvested and counted.
(TIF)

Table S1 **PCR primers used to detect mouse gene expressions.**
(PDF)

Table S2 **Comparison of albumin secretion levels among different culture conditions.**
(TIF)
