# Supplementary figures and images for: Efficient Differentiation of Embryonic Stem Cells into Hepatic Cells In Vitro Using a Feeder-Free Basement Membrane Substratum
Source: PLoS One. 2011 Aug 26;6(8):e24228. doi: 10.1371/journal.pone.0024228 (PMC3162614; doi:10.1371/journal.pone.0024228)

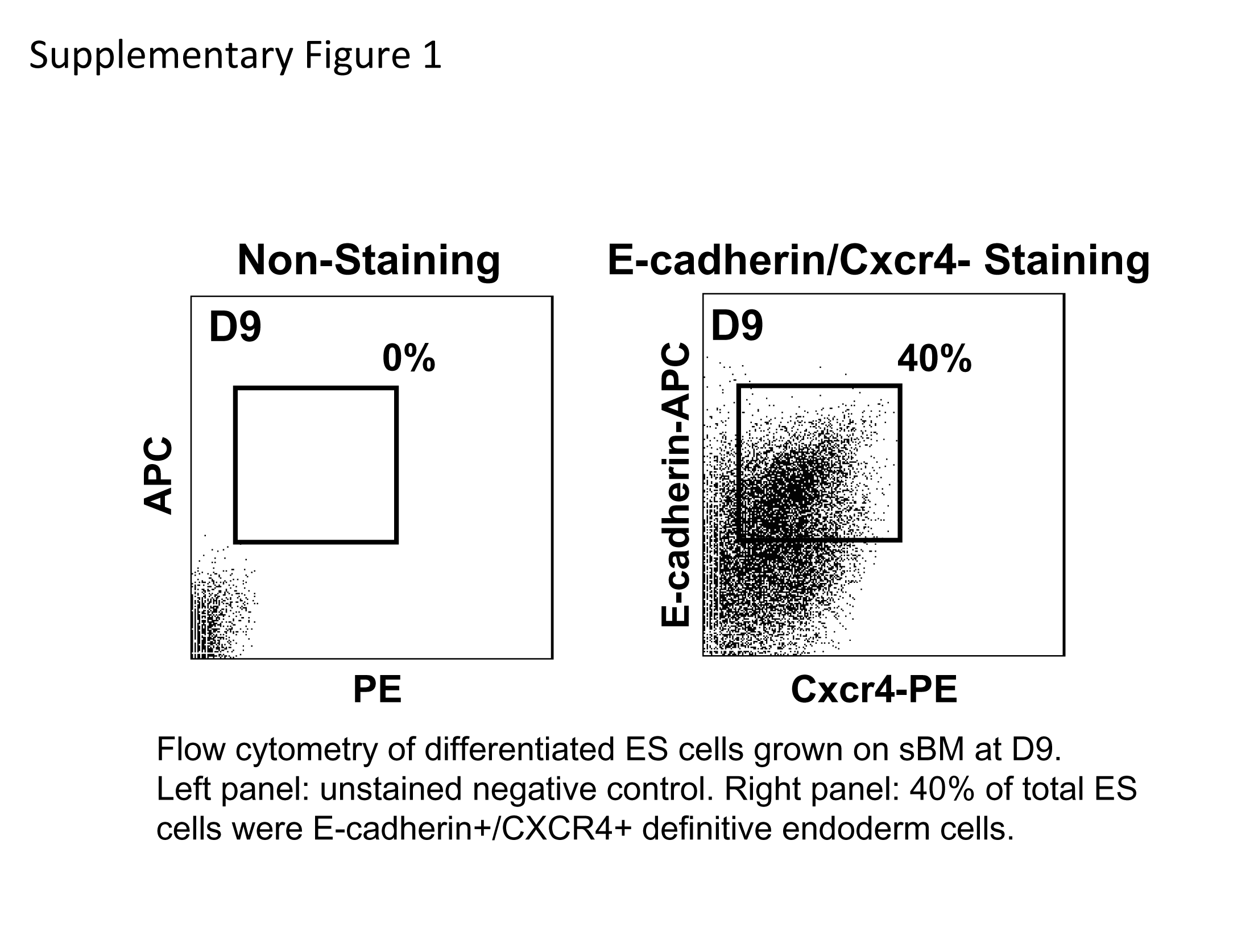

Supplement: Figure S1 — Flow cytometry of differentiated ES ells grown on sBM on D9. Left panel: unstained negative control. Right panel: 40% of total ES cells were E-cadherin+/CXCR4+ definitive endoderm cells. (TIF) [file pone.0024228.s001.tif]

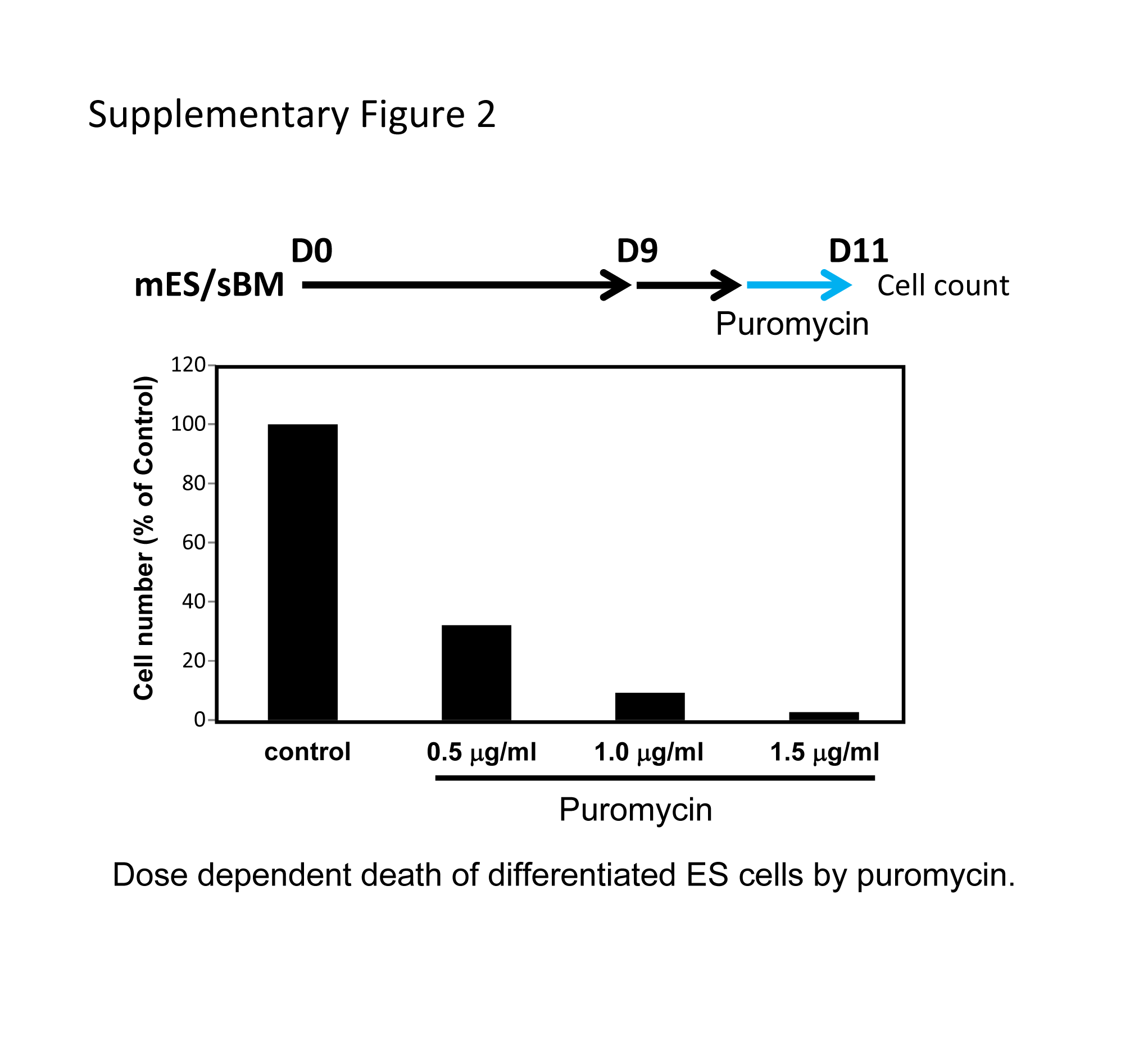

Supplement: Figure S2 — Dose dependent death of differentiated ES cells by puromycin. ES cells were cultured as described in Fig. 1 and 0.5, 1.0 or 1.5 µg/ml puromycin were added from D10 to D11. On D11, cells were harvested and counted. (TIF) [file pone.0024228.s002.tif]

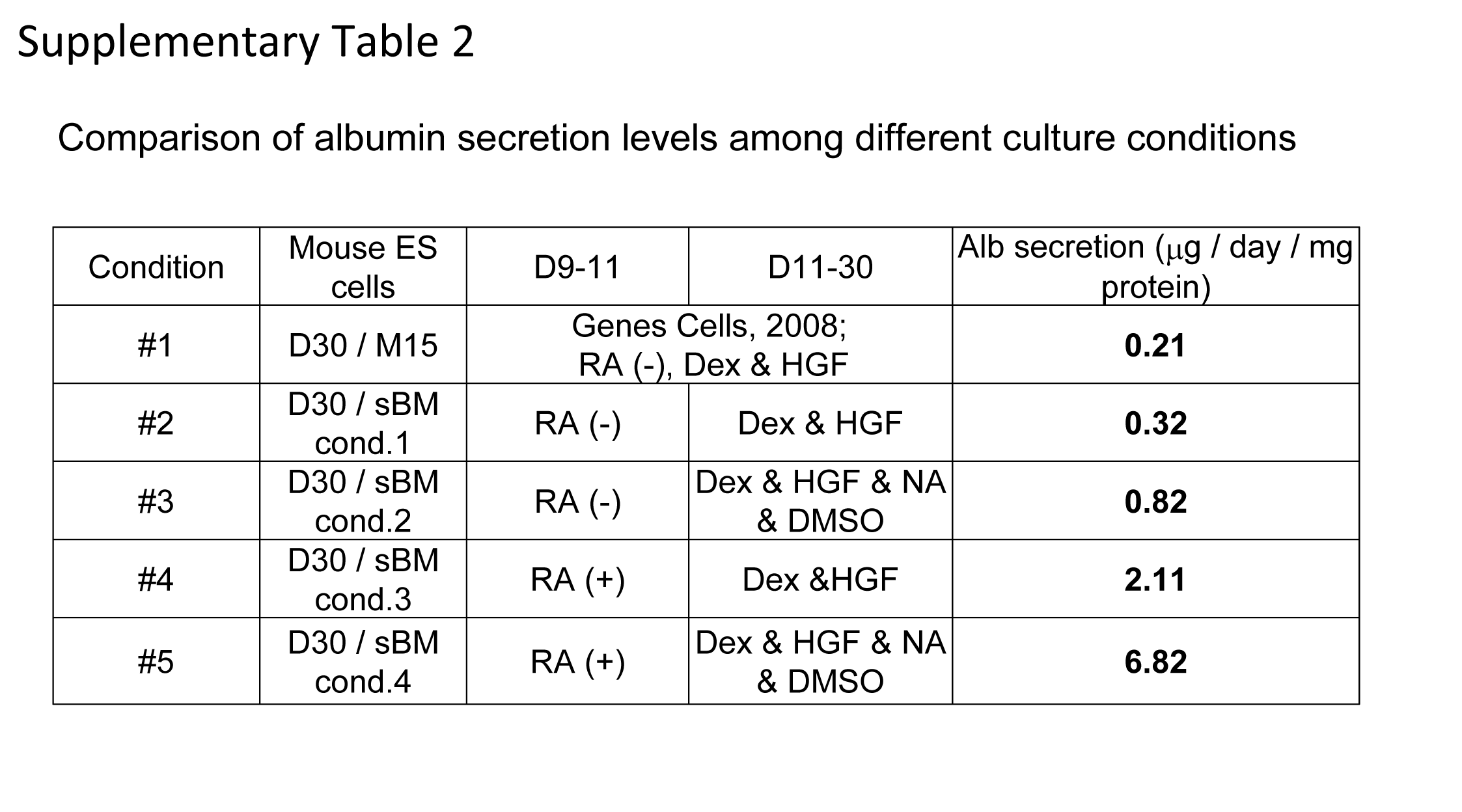

Supplement: Table S2 — Comparison of albumin secretion levels among different culture conditions. (TIF) [file pone.0024228.s004.tif]
