# Supplementary material for: Efficient Differentiation of Embryonic Stem Cells into Hepatic Cells In Vitro Using a Feeder-Free Basement Membrane Substratum
Source: PLoS One. 2011 Aug 26;6(8):e24228. doi: 10.1371/journal.pone.0024228 (PMC3162614; doi:10.1371/journal.pone.0024228)
Supplement: Table S1 — PCR primers used to detect mouse gene expressions. (PDF) [file pone.0024228.s003.pdf]

Supplementary Table 1. PCR primers used to detect mouse gene expressions

Gene	Forward primer	Reverse primer
mouse		
αat	TCGATCCTAAGCACACTAAGG	CGGCTTGTAAGACTGTAGC
Afp	TCGTATTCCAACAGGAGG	AGGCTTTTGCTTCACCAG
Alb	CTTAAACCGATGGGCGATCTCACT	CCCCACTAGCCTCTGGCAAAAT
β-actin	GTGATGGTGGGAATGGGTCA	TTTGATGTCACGCACGATTTCC
Cyp3a11	ATAGAGCTTTGCTGTCCCCC	CGGCTTTCCTTCATTCTGTC
Cyp7a1	CAACGGGTTGATTCCATACC	ATTTCCCCATCAGTTTGCAG
Foxa2	TGGTCACTGGGGACAAGGGAA	GCAACAACAGCAATAGAGAAC
Gsc	GCACCATCTTCACCGATGAG	AGGAGGATCGCTTCTGTCGT
Hex	GTTCTCCAACGACCAGACCG	GGAGGGTGA ACACTGCGAAC
Hnf4a	ACACGTCCCCATCTGAAGGTG	CTTCCTTCTTCATGCCAGCCC
Hnf6	CCGGAGTTCCAGCGCAT	TCTTGCTCTTTCCGTTTGCA
Itgb1	TTGGGATGATGTCGGGAC	AATGTTTCAGTGCAGAGC
Mixl1	GCACGTCGTTCAGCTCGGAGCAGC	AGTCATGCTGGGATCCGGAACGTGG
Oct3/4	GAGGAAGCCGACAACAATGAGAACCTTCAG	TTCTGGCGCCGGTTACAGAACCATACTCGA
Sox17	GAACAGTTGAGGGGCTACAC	GTTTAGGGTTTCTTAGATGC
T	ATGCCAAAGAAAGAAACGAC	AGAGGCTGTAGAACATGATT
Human		
Afp	TGCCAACTCAGTGAGGACAA	TCCAACAGGCCTGAGAAATC
Alb	GATGTCTTCCTGGGCATGTT	ACATTTGCTGCCCACTTTTC
Cyp3a4	CAGGAGGAAATTGATGCAGTTTT	GTCAAGATACTCCATCTGTAGCACAGT
Cyp7a1	AATTCCATACCTGGGCTGTG	AGGCAGCGGTCTTTGAGTTA
Gapdh	CGAGATCCCTCCAAAATCAA	CATGAGTCCTTCCACGATACCAA
Ntcp	GGAGGGAACCTGTCCAATGTC	CATGCCAAGGGCACAGAAG
Oatp2b1	CTTCATCTCGGAGCCATACC	GCTTGAGCAGTTGCCATTG
Sult2a1	TCGTCATAAGGGATGAAGATGTAATAA	TGCATCAGGCAGAGAATCTCA
Ugt1a1	ATGCTGTGGAGTCCCAGGGC	CCATTGATCCCAAAGAGAAAACC

αat, α-antitrypsin; Afp, α-fetoprotein; Alb, albumin; Itgb1, integrin, beta 1; Gsc, Goosecoid; Oatp2b1, organic anion transporter family, member 2b1; Ntcp, Sodium-taurocholate cotransporting polypeptide; Sult2a1, sulfotransferase family 2A dehydroepiandrosterone-preferring member 1; Ugt1a1, UDP glucuronosyltransferase 1 family polypeptide A1
